# USPIO enhanced MR imaging in CNS tumors (UMIC): a study protocol

**DOI:** 10.1080/17435889.2026.2681555

**Published:** 2026-06-10

**Authors:** J. Breese, W. Lloyd, A. Fothergill, I. Djoukhadar, A. Tyler, E. Bullock, K. Cloran, K. Li, X. Zhu, B. Alfaifi, T. Sereno, P. Naveen Golchha, A. McMahon, K. Karabatsou, P. D’Urso, H. Maye, M. Bailey, C. Hannan, A.T. King, A. Jackson, R. Hinz, D. Lewis, D. Coope

**Affiliations:** a Division of Informatics, Imaging and Data Sciences, School of Health Sciences, Faculty of Biology, Medicine and Health, University of Manchester, Manchester, UK; b Geoffrey Jefferson Brain Research Centre, Manchester Centre for Clinical Neurosciences, Northern Care Alliance NHS Foundation Trust, Manchester, UK; c Division of Human Communication, Development & Hearing, University of Manchester, Manchester, UK; d Department of Chemistry, School of Natural Sciences, Faculty of Science and Engineering, University of Manchester, Manchester, UK; e Division of Neuroscience, School of Biological Sciences, Faculty of Biology, Medicine and Health, University of Manchester, Manchester, UK; f Division of Cancer Sciences, School of Health Sciences, Faculty of Biology, Medicine and Health, University of Manchester, Manchester, UK

**Keywords:** Ferumoxytol, glioma, magnetic resonance imaging, tumor-associated macrophages, ultrasmall superparamagnetic iron oxide nanoparticles, vestibular schwannoma

## Abstract

Tumor-associated macrophages (TAMs) are key drivers of brain tumor progression and therapy resistance. Clinically applicable biomarkers capable of evaluating TAM populations *in vivo* are highly sought, with one potential imaging marker being ultrasmall superparamagnetic iron oxide nanoparticle (USPIO)-enhanced MRI. Ferumoxytol is a commercially available, now FDA-approved USPIO contrast agent, which circulates for up to 24 hours before undergoing extravasation and TAM-mediated phagocytosis. This study investigates the feasibility of USPIO-enhanced MRI as a noninvasive marker of TAM-associated inflammation in suspected transforming gliomas and vestibular schwannoma (VS). Patients undergo MRI at four timepoints over three days. Following an initial gadolinium-based MRI protocol, participants receive a slow ferumoxytol infusion (5 mg/kg, max 510 mg), with imaging performed immediately post-infusion and at 24 and 48 hours. Tumor tissue obtained at surgery is then analyzed to determine the cellular localization of USPIO internalization. This pilot study aims to characterize USPIO uptake and clearance within the tumor microenvironment, define the distribution and phenotype of USPIO-internalizing TAMs, and identify optimal imaging methods for this TAM quantification. By enabling *in vivo* characterization of TAM-rich regions, this work may support USPIO-enhanced MRI as a clinical biomarker of inflammation that can inform patient selection for immunomodulatory therapies.

Trial Registration Number: NCT06572475

## Plain Language Summary

In many brain tumors, inflammatory cells called macrophages are present in high numbers and are linked to tumor growth. A key question, however, is how these macrophages can be detected, measured and monitored in the clinic. A clinically applicable imaging test that can accurately measure tumor macrophage content would be of considerable value. One such test is through ultrasmall superparamagnetic iron oxide (USPIO)-enhanced magnetic resonance imaging (MRI). Following intravenous injection, USPIO circulate in the bloodstream before being taken up by macrophages in tumor tissue, wherein they can be detected by MRI. This pilot study is to evaluate if a commercially available USPIO preparation called ferumoxytol can accurately measure macrophages in brain tumors, focusing on vestibular schwannomas and transforming gliomas. Participants will undergo dedicated MRI sequences before receiving ferumoxytol, then shortly after (<2 hours) and at both 24 and 48 hours after injection. For patients having surgery, imaging results will be compared with tumor samples so that markers of inflammation can be compared with USPIO uptake. The goal is to establish the most reliable way to measure macrophages and determine whether this MRI technique can guide the development of new inflammation-targeted treatments for brain tumors.

## 1. Introduction

### 1.1. Background and rationale

The official title of this study is: USPIO Enhanced MR Imaging in CNS Tumors (UMIC): A pilot study of ferumoxytol (Feraheme®) as an imaging biomarker of tumor-associated macrophage infiltration in vestibular schwannomas (VS) and low-grade gliomas (LGG) (Clinicaltrials.gov identifier: NCT06572475 [1]). The purpose of this single-center, prospective, observational imaging pilot study is to evaluate whether a commercially available iron oxide nanoparticle, ferumoxytol, can serve as a quantitative imaging biomarker of tumor-associated macrophage (TAM) burden in central nervous system tumors, with an initial exploratory focus on suspected transforming LGGs and VS.

#### 1.1.1. The role of TAMs in the growth and progression of CNS tumors

Low-grade gliomas (LGGs), including diffuse astrocytomas, account for approximately 10–20% of all primary brain tumors. Although they are more indolent relative to grade III (anaplastic) or grade IV tumors including glioblastomas (GBMs), over 95% of LGGs will undergo malignant transformation into a higher grade (III/IV) gliomas (HGGs) [2]. Depending on tumor location, glioma typically present with seizures, focal neurological signs, or symptoms of raised intracranial pressure and these tumors have a disproportionate impact on patients and wider society, often affecting young adults [3]. In other non-CNS cancers, TAMs are known to be pivotal in driving malignant transformation and may contribute to an LGG’s propensity for infiltration, recurrence and therapy resistance [4–7]. Currently, the appearance of focal contrast enhancement within these tumors, suggestive of loco-regional blood–brain barrier (BBB) disruption, is the most used sign of tumor progression in clinical practice. However, this lacks specificity, with the temporal relationship between histological transformation and appearance of new contrast enhancement unclear [8,9].

VS are histologically benign tumors, arising from Schwann cells lining the vestibulocochlear nerve at the cerebellopontine angle [10]. Whilst 95% occur as a unilateral, sporadic tumor, they can also occur bilaterally as part of the syndrome NF2-related Schwannomatosis (NF2-SWN) [11]. The associated morbidity of VS and their treatment (surgery or radiosurgery) makes management through surgery or radiotherapy particularly challenging [12]. *Ex vivo* VS tissue analysis studies have shown that TAMs compartmentalize within Antoni type B areas and are associated with increased tumor size and growth rate [13]. *In vivo* evidence for increased TAM-dependent inflammation with growing VS also stems from TSPO positron emission tomography (PET) studies, where inflammation in growing tumors is attributed to the abundance of TAMs [13]. Subsequent work demonstrated that TAMs are a targetable driver of the immune microenvironment and tumor growth in NF2-SWN-associated VS also [14–16].

#### 1.1.2. Imaging CNS TAMs using USPIO-enhanced MRI

**Figure 1.**
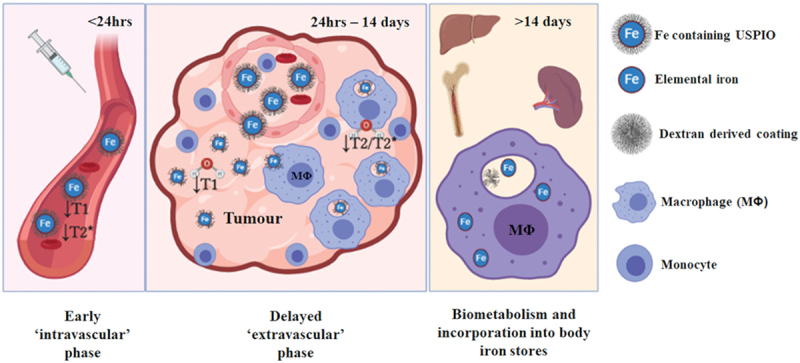
Distribution and biometabolism of USPIO following intravenous administration. Following intravenous injection, USPIO nanoparticles initially circulate within the bloodstream (<24 hours; intravascular phase), producing T_1_and T_2_* shortening. In regions of blood–brain barrier disruption, USPIO extravasate over 24–48 hours into the extravascular space and are phagocytosed by TAMs, leading to intracellular iron accumulation and delayed T_1_and T_2_/T_2_* contrast changes. At later time points (>14 days), USPIO are taken up by the mononuclear phagocyte system, with iron incorporated into systemic iron stores and nanoparticle coatings cleared from the body.

Considering the role of TAMs in tumor growth, paired with the advent of TAM–targeted cancer immunotherapies, a diagnostic tool capable of detecting, quantifying and monitoring TAM abundance *in vivo* is required. One potential tool, is through ultrasmall superparamagnetic iron oxide (USPIO) nanoparticles (<50 nm), consisting of an iron oxide core surrounded by a carbohydrate or polymer coating [17]. Ferumoxytol is a commercially available USPIO preparation that has FDA approval as an iron replacement therapy and most recently as an MRI contrast agent (Feraheme®, Azurity Pharmaceuticals) [18,19]. The iron USPIO core of ferumoxytol induces strong T_1_ and T_2_/T_2_* relaxation effects, and its size and coat results in a long circulating half-life following intravenous administration (ferumoxytol, t½ = 14–21 hours) [17,20]. Early phase USPIO imaging (<24 hours post-injection) allows for high-resolution MRI angiography whilst circulating in the vasculature. In areas of endothelial disruption, such as within CNS tumors, USPIO slowly (~24–48 hours) extravasates into the tissue extravascular-extracellular space (EES), showing delayed signal enhancement on T_1_-weighted imaging [21]. Ferumoxytol is rapidly phagocytosed by TAMs, as outlined in Figure 1 [22–24]. The engulfment and focal concentration of USPIO within TAMs creates local magnetic susceptibility effects, reducing signal intensity on T_2_* and susceptibility-weighted MRI acquisitions.

Early studies utilizing USPIO in WHO grade IV gliomas demonstrated additional areas of enhancement on delayed imaging, when compared to standard post-GBCA (gadolinium-based contrast agent) sequences [22]. More recently, post-treatment studies in HGGs undergoing chemoradiotherapy have demonstrated that delayed USPIO-enhanced MRI can differentiate pseudoprogression (transiently increased volumes of contrast enhancement within the radiation field) from true disease progression in IDH-1 (isocitrate dehydrogenase 1) wild-type and IDH-1 mutated tumors [25]. Whilst a growing number of studies have investigated USPIO-related enhancement and uptake in HGGs, no studies have investigated the utility of these clinically available USPIO compounds in diffuse or transforming LGGs [7,25–28]. Therefore, it is unclear how the lesser degree of BBB disruption, and the immune phenotype in these tumors (compared to HGGs), will impact both extravasation of USPIO and its cellular internalization. Whilst early studies have reported isolated cases of USPIO uptake in histologically benign, extra-axial tumors such as meningioma there also remains a paucity of studies in non-astrocytic, extra-axial tumors such as VS that sit outside the blood–brain barrier [29].

#### 1.1.3. Cellular source of USPIO internalization in vivo

The timing and mechanisms of USPIO trafficking into the brain and the exact cell-type specificity are yet to be fully understood. Previous *in vivo* models have demonstrated ferumoxytol localization within the lysosome of tumor CD68^+^/CD163^+^tumor macrophages, and within dilated astroglial end feet and astroglial processes in the parenchyma [30]. Data from human studies is limited to HGGs, and have shown iron deposition within CD68^+^/CD163^+^ TAMs following ferumoxytol (5 mg/kg) infusion, with significant positive correlations between delayed imaging and the number of CD68^+^/CD163^+^ iron-containing TAMs at time of tumor resection [26]. More recently, a study of 10 patients with IDH-wildtype GBMs, demonstrated that tissue samples from ferumoxytol enhancing regions contained significantly more M2 polarized macrophages than non-ferumoxytol enhancing tissue regions [28]. However, these studies are limited to high-grade tumors only, do not fully define the temporal relationship between vascular extravasation and intracellular uptake, nor assess their relative contributions to the evolving MRI signal. There is, therefore, a clear need for larger patient studies of USPIO-enhanced imaging in CNS tumors with comparative tissue datasets, to clarify the cellular source of the USPIO-enhanced MRI signal and evaluate the *in vivo* relationship between BBB disruption and USPIO extravasation.

### 1.2. Objectives

In this pilot study, we investigate the candidate USPIO agent ferumoxytol as a noninvasive imaging marker of TAM-driven inflammation in suspected transforming LGGs and VS. We hypothesize that delayed ferumoxytol-sensitive MRI signals reflect the transition from vascular circulation to extravascular and TAM-associated USPIO uptake within the tumor microenvironment.

The primary endpoint is to assess the association between delayed ferumoxytol-induced MRI susceptibility changes and histologically quantified TAM density, across CNS tumor subtypes with differing vascular and microenvironmental characteristics. Within this, the specific secondary endpoints are to:

- Characterize the volume, pattern, and intensity of early and delayed USPIO-enhanced MRI signal in gliomas and VS and define the optimal qualitative (e.g., susceptibility-weighted imaging, SWI) and quantitative methods (e.g., quantitative susceptibility mapping, QSM and T2* mapping) for assessing delayed USPIO cellular internalization.
- Examine the relationship between GBCA enhancement patterns and the extent, spatial distribution and signal intensity of early (<2 hours) and delayed (24–48 hours) USPIO uptake in both glioma and VS cohorts.
- Examine the association between delayed USPIO-enhanced MRI signal, tissue markers of TAM abundance and VS clinical behavior, such as size and VS growth status.
- Evaluate QSM-derived susceptibility values as a quantitative imaging marker for detecting USPIO cellular internalization in CNS tumors, and the relationship with tissue measures of iron abundance and localization.
- Assess differences in USPIO-related signal changes and immune cell density between histologically confirmed transformed and non-transformed glioma regions
- Evaluate the phenotype and spatial distribution of TAMs in each tumor and their relationship to USPIO uptake

Our specific hypotheses include that: (1) early (<2 hours) USPIO-enhanced imaging will principally reflect vascular distribution and will correspond with other MRI-derived measures of tumor vascular density and perfusion; (2) delayed (24–48 hours) USPIO-enhanced imaging will principally reflect intracellular USPIO distribution within TAM, and will co-localize with but also extend beyond imaging (GBCA enhancement) and tissue markers of endothelial disruption; (3) that QSM-derived susceptibility estimates and T2*-based measures will provide optimal quantitative reliable measures of TAM density, iron abundance and cellular iron localization with imaged tumors; (4) susceptibility based measures of delayed USPIO uptake (e.g., QSM, R2* mapping) will be greater in growing VS, relative to non-growing tumors, and will reflect an increased abundance of TAM in these tumors; and (5) that regions of higher grade transformation within imaged glioma will show changes on delayed USPIO-enhanced acquisitions, in particular susceptibility-based measures such as QSM, and these regions will show greater TAM abundance on matched tissue sections compared to non-enhancing tumor regions.

### 1.3. Trial design

Single-center, prospective, observational imaging pilot study

## 2. Methods

### 2.1. Study setting

Single-center study being undertaken at the Greater Manchester Neurosciences Center.

### 2.2. Eligibility criteria

Two main tumor groups of participants will be recruited for this study.

Group A: Patients with either sporadic or NF2-SWN-associated VS. The sample will include growing tumors that are being considered for surgical resection (sporadic tumors) or treatment with the anti-angiogenic agent bevacizumab (NF2-SWN-associated VS), and non-growing/static tumors that are being considered for either further radiological surveillance or surgery.

Group B: Patients with a suspected transforming LGG. Patients in this cohort will have a suspected diagnosis of an LGG on conventional clinical imaging, with or without radiological features suggestive of malignant transformation toward either grade III (anaplastic) or grade IV gliomas. Patients who are listed to undergo surgical debulking or biopsy will be enrolled into the study so that acquired imaging can be compared with tissue datasets.

*Inclusion criteria*:

- Be at least 16 years old
- Have a CNS tumor suspected to be one of the defined histological types
- Be able to lie still for up to 1 hour comfortably

*Exclusion criteria*:

- Life expectancy of <1 year
- Previous CNS radiotherapy/stereotactic radiosurgery (SRS)
- Pregnancy/ breastfeeding in females
- An eGFR of <30 ml/min
- A documented history of iron overload/hemosiderosis/hemochromatosis
- Presence of other immune or inflammatory conditions e.g., rheumatoid arthritis
- Absolute (e.g., pacemaker) or relative (severe anxiety or claustrophobia) contraindications to MRI scanning
- A history of allergic reaction to iron or dextran
- A history of allergic reaction to gadolinium contrast agents, asthma or renal problems
- Inability to understand verbal explanations or written information given in English.

### 2.3. Interventions

#### 2.3.1. Imaging protocol

**Figure 2.**
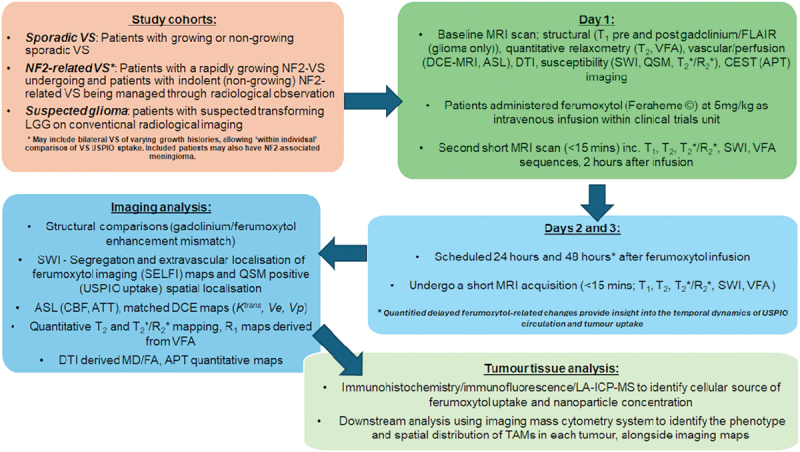
Study design and scanning workflow.

Patients are recruited via specialist multi-disciplinary team meetings at the Greater Manchester Neurosciences Center. Recruited patients are then scanned at four timepoints over three days. A flowchart of participant activities is shown in Figure 2.

ASL, arterial spin labeling; ATT, arterial transit time; APT, amide proton transfer; CEST, chemical exchange saturation transfer; CBF, cerebral blood flow; DCE-MRI, dynamic contrast-enhanced MRI; DTI, diffusion tensor imaging; FA, fractional anisotropy; FLAIR, fluid-attenuated inversion recovery; *K*^*trans*^, volume transfer constant; LA-ICP-MS, laser ablation inductively coupled plasma mass spectrometry; MD, mean diffusivity; QSM, quantitative susceptibility mapping; R_1_, longitudinal relaxation rate (1/T_1_); R_2_*, effective transverse relaxation rate (1/T_2_*); SELFI, susceptibility-based enhanced longitudinal ferumoxytol imaging; SWI, susceptibility-weighted imaging; T_1_, longitudinal (spin–lattice) relaxation time; T_2_, transverse (spin–spin) relaxation time; T_2_*, effective transverse relaxation time;*Ve*, extravascular extracellular volume fraction; VFA, variable flip angle; *Vp*, plasma volume fraction; USPIO, ultra-small superparamagnetic iron oxide.

On the first visit, patients undergo a baseline advanced MRI protocol at 3T, including: structural T_1_W and T_2_ FLAIR imaging (glioma only), baseline T_1_ relaxivity (R_1_) mapping (measured using a variable flip angle acquisition), SWI, T_2_ and T_2_* mapping, QSM, and DTI. Amide proton transfer (APT) imaging, a chemical exchange saturation transfer MRI technique that is sensitive to changes in proteins and peptides containing amide groups in tissue, is also performed for recruited glioma patients. It is not performed for participants with VS, as its application in the inferior brain and skull base is often limited by artifacts arising from B_0_ field inhomogeneities at air–tissue interfaces, particularly near the paranasal sinuses and external auditory canals [31]. For measuring tumor perfusion and tumor microvascular characteristics (including endothelial permeability) a multi-delay ASL acquisition and a dual-injection, dual–temporal resolution DCE-MRI acquisition is also collected. During the DCE-MRI protocol, administration of a GBCA (gadoterate meglumine; Dotarem®, Guerbet S.A.; dose of 0.2 mmol/kg) is performed, and is followed by post-contrast T1W imaging and repetition of the pre-contrast T_1_, T_2_ and T_2_* mapping, SWI, and QSM protocols. An overview of included sequences and acquisition parameters are provided in Supplementary Table S1.

After the initial baseline MRI scan, patients receive ferumoxytol at a dose of 5 mg/kg (up to maximum dose of 510 mg) as a slow intravenous infusion over 30 minutes. Patient observations (blood pressure, heart rate, temperature) are performed regularly during the infusion and up to 30 minutes afterward, due to a small risk of serious hypersensitivity reactions following IV iron infusions.

Scan 2 is performed within two hours of the ferumoxytol infusion, and incorporates the same T_1_W imaging, T_1_ relaxivity (R_1_) mapping, SWI, T_2_ and T_2_* mapping and QSM protocol used for the baseline scan. This short (<20 minutes) imaging protocol allows for the evaluation of ferumoxytol-related alterations in brain and tumor vasculature at this early timepoint. Visits 2 and 3 (scans 3 and 4) occur 24 and 48 hours, capturing the expected peak of USPIO uptake in TAMs post-infusion. During these subsequent visits, patients undergo the same brief MRI protocol as conducted at scan 2. This will enable the assessment of delayed (>24 hours) ferumoxytol-related imaging changes relative to baseline.

Since participants receive both GBCA and ferumoxytol, potential interactions between contrast-agent effects will be considered during image interpretation. Prior protocol optimization phantom experiments performed by our group, which demonstrated how GBCA is expected to predominantly influence T_1_-weighted and R_1_ measurements, whereas ferumoxytol is expected to exert stronger effects on T_2_, T_2_*/R_2_/R_2_*, SWI and QSM-derived susceptibility measures (unpublished). Accordingly, GBCA-enhanced imaging will primarily be used to define conventional enhancement and permeability-related tumor regions, while delayed ferumoxytol assessment will prioritize T_2_, T_2_*/R_2_/R_2_*/SWI and QSM-derived measures. The acquisition of R_1_, R_2_, R_2_*, T_2_*, T_2_, SWI and QSM data across pre-contrast, post-GBCA, early post-ferumoxytol and delayed post-ferumoxytol timepoints will also allow the *in vivo* signal effects of each contrast agent to be assessed across complementary quantitative imaging markers.

### 2.4. Tissue collection

Written informed consent is obtained from all participants for the collection and analysis of tumor tissue, with both formalin-fixed paraffin-embedded (FFPE) and fresh-frozen specimens collected at surgery. Immunohistochemistry and immunofluorescence analyses are performed on FFPE and fresh-frozen tissue to characterize USPIO uptake across immune and nonimmune cell populations. For extra-axial tumors such as VS, tumors are often completely resected allowing comparison of ferumoxytol imaging datasets with tissue across the entire tumor volume. For intra-axial tumors such as glioma, image-guided tissue sampling of non-enhancing and enhancing regions on delayed phase (>24 hours) post ferumoxytol imaging can be performed using neuronavigation software.

The interval between the final ferumoxytol MRI scan and tissue acquisition will be recorded. Surgery will be scheduled as close as clinically feasible to the final imaging timepoint, determined by clinical urgency, theater availability and standard care pathways. In line with the usual clinical pathway at our institution, for participants with glioma, it is expected, that surgery will be performed within 24 hours of the final MRI scan. For participants with VS, it is expected that surgery will be performed within 4 weeks of the final MRI scan. Acquired imaging from each timepoint (post GBCA and post ferumoxytol) will be fused using neuronavigation software. For participants with glioma, sampling will target regions within the tumor, where safe and appropriate to do so, corresponding to delayed ferumoxytol related changes, contrast enhancement and non-enhancing tumor. For participants with VS, complete or near-complete resection is standard practice at our institution but we will also use neuronavigation to allow for tissue sampling of different regions within the tumor. Sample location will be recorded for later comparison with MRI-derived maps. Imaging–histology registration will be interpreted with recognition of unavoidable spatial uncertainty arising from brain shift, tissue deformation, sampling limitations, fixation and sectioning effects, and differences between *in vivo* MRI resolution and *ex vivo* histological resolution.

### 2.5. Outcomes

#### 2.5.1. Post-scan processing

Post-contrast T_1_-weighted images acquired following both gadolinium-based contrast agent administration and delayed ferumoxytol imaging (24–48 hours post-injection) will be used to delineate tumor regions of interest (ROI). Tumor segmentation will be performed using semi-automatic approaches in ITK-SNAP [32,33]. Diffusion tensor imaging (DTI) data will be processed to derive fractional anisotropy (FA) and mean diffusivity (MD) maps following eddy-current and motion correction. Multi-delay arterial spin labeling (ASL) will be used to generate quantitative cerebral blood flow (CBF) maps. Amide proton transfer (APT) imaging will be processed to obtain magnetization transfer ratio asymmetry maps. Susceptibility-weighted imaging (SWI), DCE-MRI, and additional quantitative MRI modalities will be processed using established pipelines as described below.

Dual-injection dual-temporal-resolution DCE-MRI data will be analyzed using the LEGATOS method [34] to derive high-spatial-resolution maps of the following key microvascular parameters: volume transfer constant (*K*^*trans*^), plasma volume fraction (_*Vp*_) and extravascular extracellular volume fraction (_*Ve*_). SWI data acquired at each timepoint will be analyzed using the ‘Segregation and Extravascular Localization of Ferumoxytol Imaging’ (SELFI) formula, which aims to characterize USPIO nanoparticle intra/extravascular compartmentalization [27]. SWI magnitude images acquired at pre-contrast, early post-ferumoxytol, and delayed post-ferumoxytol time points will be co-registered to non-contrast-enhanced T_1_-weighted images. Voxel-wise logarithmic signal intensity ratios will be calculated between pre-contrast and ‘early’ SWI maps or delayed post-contrast images to estimate relative USPIO concentrations. Subtraction of delayed from early ratio maps will yield positive SELFI^+^ and negative SELFI^–^ values. SELFI^+^ values are thought to reflect extravascular USPIO uptake localized to phagocytic cells (TAMs), whereas SELFI^–^ values indicate persistent intravascular USPIO signal, consistent with regions of higher microvascular density [27]. SELFI values will be compared with maps of quantitative relaxometry values (T_2_*, T_2_) generated using existing pipelines and QSM-derived susceptibility maps. As the SELFI method is an investigational approach and not yet an established clinical biomarker, it may be influenced by image registration error, susceptibility artifact, baseline hemorrhage or calcification, residual intravascular USPIO, and nonlinear signal behavior on SWI magnitude images. SELFI maps will therefore be interpreted alongside QSM, T_2_*, SWI and tissue-based measures rather than as a stand-alone marker of cellular USPIO uptake.

During the study, a QSM protocol for visualizing absolute local magnetic field susceptibility changes resulting from cellular USPIO compartmentalization, will be optimized and evaluated. QSM differs from traditional hypo-intensity contrast in SWI or T_2_*, reflecting local rather than regional tissue susceptibility, overcoming blooming artifacts that arise through incorporating a dipole deconvolution during processing [35,36]. QSM data will be analyzed using the Morphology Enabled Dipole Inversion (MEDI) toolbox (v.2020), a widely validated pipeline for processing complex gradient echo data [36,37]. Early timepoint QSM maps are expected to reflect susceptibility shifts predominantly related to intravascular USPIO, whereas delayed maps (24 and 48 hours post-injection) are hypothesized to capture both residual intravascular USPIO and susceptibility effects, attributable to extravascular uptake by TAMs. Susceptibility values and spatial distribution patterns will be quantified across timepoints and evaluated alongside other multimodal maps, including DCE-MRI parameters and tissue data, to further explore the relationship between susceptibility changes, TAM infiltration and microenvironment features such as permeability and perfusion.

### 2.6. Tissue analysis

Resected specimens will be analyzed using established immunohistochemical protocols [14,38]. FFPE sections will be stained to quantify TAM density (Iba1), vascular features (CD31, fibrinogen), and Schwann cell markers (CD56). Ferumoxytol deposition within resected tissue will be assessed using Perls’ Prussian blue staining for iron and immunolabelling of the dextran ferumoxytol coating (Dx1). The cellular localization of ferumoxytol uptake will be further characterized using dual immunohistochemistry and immunofluorescence. Laser ablation inductively coupled plasma mass spectrometry (LA-ICP-MS) will also be used to detect and spatially map iron derived from USPIO uptake within resected tumor tissue [39–41]. To evaluate in detail the phenotype and spatial distribution of TAMs in each tumor and their relationship to USPIO uptake, selected tissue specimens will be analyzed using imaging mass cytometry and a previously validated antibody panel [11,42].

### 2.7. Sample size and tumor groups

The planned number of participants is 39 (12 sporadic VS, 12 NF2-VS patients, 15 glioma patients). This sample size reflects feasibility and the expected recruitment capacity of a single-center pilot study requiring serial advanced MRI over three days, ferumoxytol infusion, and, in surgical participants, tissue acquisition for imaging-histology comparison. Although glioma and VS are biologically distinct, both are characterized by macrophage-rich inflammatory tumor microenvironments that are implicated in tumor growth, progression and treatment response. The inclusion of both intra-axial gliomas and extra-axial VS permits assessment of our hypotheses across two clinically important CNS tumor settings with differing characteristics.

This pilot study will allow the evaluation of recruitment feasibility, scan completion rates, tolerability, missing data rates, USPIO-sensitive MRI metrics, and preliminary effect sizes for imaging-histology associations. Subgroup analyses by tumor type, VS growth status and glioma transformation status will therefore be interpreted as exploratory, to inform future larger-scale studies.

### 2.8. Data collection, management, and analysis

Multimodal imaging analysis will assess the relationships between USPIO uptake and tumor vascular, microstructural, and inflammatory characteristics. Spatial associations will be examined between ferumoxytol-sensitive imaging markers (T_2_, T_2_*, SWI and SELFI values, QSM derived susceptibility values), ASL and DCE-MRI–derived microvascular parameters (*K*^*trans*^, *Ve, Vp*, blood flow), and diffusion metrics (mean diffusivity and fractional anisotropy) using voxel-wise and region-of-interest–based analyses. Early and delayed post-ferumoxytol imaging will be compared to assess time-dependent USPIO redistribution across vascular and extravascular compartments. In patients undergoing surgical resection, imaging-derived measures of USPIO uptake will be correlated with tissue-based markers of inflammation and microvascular proliferation. Spatially resolved elemental iron maps derived from LA-ICP-MS will be compared with adjacent histological sections to support spatial validation of imaging-defined USPIO uptake and compared with corresponding MRI-derived USPIO-sensitive metrics.

### 2.9. Statistical analysis

Differences between ROI volumes on post-GBCA T_1_-weighted and delayed (24–48 hour) USPIO-enhanced T_1_-weighted images will be evaluated using overlap measures, the Dice similarity coefficient (DSC) and Jaccard similarity coefficients (JSC), and the Structural Similarity Index Measure (SSIM) [43,44].

To satisfy primary endpoints, the associations between imaging-derived and tissue-derived parameters will be assessed using Pearson’s product-moment correlation coefficient for approximately normally distributed variables, or Spearman’s rank correlation coefficient for non-parametric or non-linear associations. Secondary analyses will assess temporal changes in imaging metrics, spatial overlap between GBCA and delayed ferumoxytol signal, and associations between USPIO-sensitive metrics and DCE-MRI, ASL and DTI-derived parameters. Comparisons between tumor subgroups, including glioma subtype and VS growth status, will be performed using t-tests or ANOVA where assumptions are met, or suitable non-parametric alternatives where appropriate [14]. These subgroup analyses will be exploratory and interpreted as hypothesis-generating.

Temporal changes in voxel-wise and whole tumor ROI imaging metrics (T_2_, T_2_*, R_1_, QSM-derived susceptibility values) within each tumor voxel will be evaluated using a repeated-measures ANOVA with Greenhouse–Geisser correction for non-sphericity and repeated-measures mixed-effect modeling [45]. Mixed-effects modeling will be preferred where repeated-measures datasets are incomplete. To address multiplicity, analyses will be pre-specified by endpoint hierarchy. For ROI-based secondary analyses involving multiple imaging metrics, false discovery rate correction or Holm–Bonferroni correction will be applied as appropriate. Voxel-wise analyses will be treated as exploratory and will use spatially appropriate correction methods, such as cluster-level correction or false discovery rate correction, where feasible. Findings from these exploratory analyses will be interpreted as hypothesis-generating, rather than confirmatory.

### 2.10. Monitoring

Recent large, multicenter registry studies indicate that when given as a slow infusion, USPIO has a favorable side-effect profile compared to the more commonly used GBCA compounds [21], with adverse reactions occurring in <2% of administrations [19]. Any adverse events thought to be related to the ferumoxytol infusion will be recorded within the case report form for each participant. Any symptoms recognized as potential side effects of parenteral iron infusion (acute myalgia, arthralgia, headache, chest pressure, and/or back pain) will be documented along with the duration of symptoms, time of onset post start of infusion and any treatment required.

Definition of serious adverse events (SAE) will be in line with NHS REC/HRA guidance on non-CTIMPs [46]. Wheezing, stridor, periorbital edema, or persistent hypotension occurrence following ferumoxytol infusion are thought to be very rare. Any such reactions to ferumoxytol (severe enough to warrant treatment with intravenous fluids and/or methylprednisolone) will therefore be classed as an SAE.

## 3. Ethics and dissemination

This study has undergone an internal review process by the sponsor within the Northern Care Alliance NHS Foundation Trust and ethical approval for the study has been granted by the North West – Liverpool Central Research Ethics Committee (REC reference 22/NW/0384). The research proposal has also undergone review by the internal scientific review committee of the pharmaceutical company supplying the ferumoxytol preparation (AMAG pharmaceuticals/Azurity pharmaceuticals) who have approved the application and the donation of Feraheme® free of charge.

## 4. Conclusion

The UMIC study presents a comprehensive multimodal framework, to evaluate USPIO-enhanced MRI as an *in vivo*, TAM-sensitive imaging biomarker of inflammatory tumor biology in glioma and VS.

This study may provide insights into the pharmacokinetics of USPIO use for *in vivo* CNS tumor imaging and for uncovering unique tumor characteristics during transformation/growth. By capturing the dynamic vascular circulation to extravascular (intracellular compartmentalization) transition spatially, this approach enables characterization of USPIO delivery, retention, and cellular uptake within heterogeneous tumor microenvironments. This may help in uncovering the mechanisms of tumor-related blood–brain barrier disruption, microvascular permeability and its spatial distribution, relative to areas of USPIO accumulation at different imaging timepoints. The multimodal imaging protocol and subsequent analyses can offer insight into tumor-specific immune biology, demonstrating how macrophage localization correlates with susceptibility-driven effects. Additionally, the mapping of TAMs beyond regions of gadolinium enhancement highlights inflammatory heterogeneity, that may not be captured by conventional gadolinium-contrast imaging. This permits the assessment of whether TAM involvement is independent of gross blood–brain barrier breakdown and/or pure vascular effects. Further, the nuanced multiparametric analysis included in this study includes the first use of QSM to assess ferumoxytol uptake in CNS tumors. This may be of significant interest to the wider neuro-oncological community, catalyzing similar studies in other tumor types and advancing understanding of how inflammation drives the progression of other conditions.

## Supplementary Material

Supplementary Table S1.pdf

## Data Availability

The data that support the findings of this study are available from the corresponding author, [DL], upon reasonable request.

## Article highlights

**Background and rationale**

- Inflammation and TAMs are key contributors to tumor growth, angiogenesis, and progression in both VS and transforming LGG.
- Current clinical imaging techniques lack the ability to visualize and quantify TAM-driven inflammation *in vivo* within the tumor microenvironment.

**Ferumoxytol and USPIO imaging**

- USPIO is preferentially internalized by TAMs and exhibits distinctive early vascular and delayed cellular MRI signal characteristics.
- When used as an off-label MRI contrast agent, ferumoxytol enables assessment of both vascular properties at early timepoints and TAM-associated uptake at delayed timepoints.
- The nanoparticle’s iron core produces measurable changes in T_1_, T_2_*, susceptibility-weighted imaging, and quantitative susceptibility mapping, allowing multiparametric characterization of tumor inflammation.

**Study design and endpoints**

- Patients with VS and suspected transforming LGGs undergo multimodal MRI before ferumoxytol administration, at early (<2 hours), and delayed (24 and 48 hours) post-injection timepoints.
- Imaging includes structural MRI, diffusion tensor imaging, DCE-MRI, susceptibility-based imaging, and relaxometry.
- Imaging-derived USPIO uptake is analyzed dynamically, using voxel-wise and region-of-interest approaches to characterize its spatial associations with tumor microvascular, microstructural, and inflammatory features and its time-dependent redistribution.
- In patients undergoing surgical resection, imaging findings are correlated with tissue-based measures of TAM density, vascular pathology, and ferumoxytol localization using immunohistochemistry, immunofluorescence, and elemental iron mapping to support validation of imaging-derived USPIO uptake.
