# Supplementary material for: USPIO enhanced MR imaging in CNS tumors (UMIC): a study protocol
Source: Nanomedicine (Lond). 2026 Jun 10;21(12):1713–21. doi: 10.1080/17435889.2026.2681555 (PMC13285558; doi:10.1080/17435889.2026.2681555)
Supplement: Supplementary Table S1.pdf [file INNM_A_2681555_SM1325.pdf]

Structural MRI acquisitions							
**Sequence**	**Cohort**	**TR (ms)**	**TE (ms)**	**Flip (°)**	**Voxel (mm³)**	**Time (min)**	**Notes**
T1w (pre/post Gd)	Both	5.91	2.7	8	1 × 1 × 1	4.2	Sequence=3D spoiled GRE (T1-TFE); Number of averages=2; Partial Fourier=Yes (phase)
T2 FLAIR	Glioma	4800	340	90	0.63 × 0.63 × 1.12	2.8	Sequence=Inversion-recovery; Number of averages=2; Inversion time (ms)=1650; Fat suppression=Yes

Vascular and perfusion MRI acquisitions							
**Sequence**	**Cohort**	**TR (ms)**	**TE (ms)**	**Flip (°)**	**Voxel (mm)**	**Time (min)**	**Notes**
ASL	Both	2675-3000	20	90	2 x 2 x 5	8.3	Partial Fourier=Yes; Echo train length=221–255; Field of view (mm^3)=224×224×150; Readout=3D GRASE; EPI factor=13; SENSE=2; Label duration (ms)=2000; PLDs (ms)=500; 1000; 1500; 2000; 2500; TR for PLDs (ms)=2575; 3075; 3575; 4075; Number of repeats=2
DCE (low-dose, high temporal)	Both	2.25	0.65	2,6,12,16	2.5 x 2.5 x 6.35	6.0	Acquisition type=3D; Partial Fourier=Yes (frequency)
DCE (full-dose, high spatial)	Both	3.00	0.92	2,6,12,16	1 x 1 x 2	5.7	Partial Fourier=Yes (frequency)

Susceptibility MRI acquisitions

Sequence	Cohort	TR (ms)	TE (ms)	Flip (°)	Voxel (mm^3)	Time (min)	Notes
QSM source (GRE phase+mag)	Both	35.40	4.1–32.1	17	0.68 × 0.68 × 1.4	2.00	Sequence=3D spoiled GRE (phase & magnitude); Echoes=5
SWI	Both	31.00	20	17	0.34 × 0.34 × 2	4.50	Sequence=3D spoiled GRE (SWI); Echoes=1; Acquisition type=3D
T2*/R2*	Both	52.29	8–50	30	0.53 × 0.53 × 2	3.30	Sequence=Multi-echo spoiled GRE; Echoes=8; Acceleration=CS ×2

Quantitative relaxometry acquisitions							
**Sequence**	**Cohort**	**TR (ms)**	**TE (ms)**	**Flip (°)**	**Voxel (mm³)**	**Time (min)**	**Notes**
T2 mapping	VS	1229	20–100	90	0.53 x 0.53 x 2	1.64	Sequence=T2 mapping (multi-echo GraSE); Echoes=5
T2 mapping	Glioma	1849	20–100	90	0.53 × 0.53 × 2	2.50	Sequence=Multi-echo GraSE; Echoes=5; Fat suppression=Yes

Diffusion MRI acquisitions

Sequence	Cohort	TR (ms)	TE (ms)	Flip (°)	Voxel (mm^3)	Time (min)	Notes
DTI	Both	2937	82.9	90	1.75 × 1.75 × 2.5	3.80	Sequence=Spin-echo EPI; b-values (s/mm^2)=0, 1000, 2000; Diffusion directions=16 + 16; b0 images=2; Phase encoding=PA

CEST MRI acquisitions

Sequence	Cohort	TR (ms)	TE (ms)	Flip (°)	Voxel (mm^3)	Time (min)	Notes
APT	Both	5929	8.3	90	1.80×1.80×6.00	3.80	Sequence=TSE-based APT-weighted; Number of slices=10; Field of view (mm^3)=230×230×72; Saturation power (µT)=2; Saturation duration (s)=2; Offsets (ppm)=-1540; ±4.3; ±3.5; ±2.7; B0 correction=Dixon
